# *Chromobacterium haemolyticum* Infection Subsequent to Experiencing a Traumatic Event in a Rice Field: A Case Report and Literature Review

**DOI:** 10.1155/crdi/6547509

**Published:** 2025-05-15

**Authors:** Mikiro Kato, Hiroyuki Kobayashi, Takuro Uchida

**Affiliations:** ^1^Department of Infectious Diseases, Mito Kyodo General Hospital, Ibaraki, Japan; ^2^Department of Internal Medicine, Mito Kyodo General Hospital, Ibaraki, Japan; ^3^Department of General Medicine, Faculty of Medicine, Juntendo University, Tokyo, Japan

**Keywords:** 16S ribosomal ribonucleic acid sequence analysis, abscess formation, beta-hemolysis, *Chromobacterium haemolyticum*, *Chromobacterium violaceum*, colony nonpigmentation, recurrence rates

## Abstract

The incidence of infections caused by *Chromobacterium haemolyticum*, phylogenetically related however distinct from *Chromobacterium violaceum*, has increased since its identification in 2008. Differences in their unique microbiological features have been highlighted, particularly regarding their phenotypic distinctions in the colony pigmentation and hemolysis. This is largely due to *C. haemolyticum* being misidentified as *C. violaceum*, using the current automated microbial identification systems. However, clinical aspects and outcomes of *C. haemolyticum* infections remain unclear as few clinically relevant cases have been reported and considered similar to *C. violaceum* infections. Consequently, we reported an extremely rare case of *C. haemolyticum* bacteremia, which was initially diagnosed as a *C. violaceum* infection, however was later confirmed to be a *C. haemolyticum* infection, using 16S ribosomal ribonucleic acid (rRNA) sequence analysis. Abscess formation was not observed, and the patient was treated with a short course of antibiotics. Ultimately, his condition resolved, without recurrence during the 1-year follow-up. Clinicians should be aware that if the isolated organism is originally identified as *C. violaceum*, however is phenotypically mismatched with colony nonpigmentation and beta-hemolysis; the organism may be *C. haemolyticum*. Mortality, abscess formation, and recurrence rates are lower than those of *C. violaceum*, and chronic broad-spectrum antibiotic suppression may not be required, potentially avoiding unnecessary antibiotic use and preventing multidrug resistance.

## 1. Introduction

*Chromobacterium haemolyticum* is a Gram-negative bacterium primarily found in the water and soil of tropical and subtropical ecosystems [1]. This organism is one of 14 species of the genus *Chromobacterium* [2, 3]. Initially, upon its identification in clinical samples, the association of *C. haemolyticum* with human diseases was not well understood. However, it has recently been recognized as a pathogen in extremely rare instances of human infections [4, 5].

Before the discovery of *C. haemolyticum*, this bacterium was considered the same as *Chromobacterium violaceum*, another bacterium that has been recognized as a rare but lethal opportunistic pathogen in humans [6]. Since C. *haemolyticum* was found to be phylogenetically related to, but different from, *C. violaceum*, the differences between them have been debated. In particular, microbiological differences in colony pigmentation and hemolysis were highlighted. This is because *C. haemolyticum* may be misidentified as *C. violaceum* using the current automated microbial identification systems. However, the clinical aspects and outcomes of *C. haemolyticum* infection are yet to be elucidated because of its rarity, and only seven clinically relevant cases have been reported [4, 5, 7–11], although it is considered similar to *C. violaceum* infection.

Here, we report an extremely rare case of *C. haemolyticum* bacteremia. The organism was initially identified as *C. violaceum*; however, colony pigmentation and hemolysis were inconsistent with the features of *C. violaceum* and was later identified as *C. haemolyticum* using 16S ribosomal ribonucleic acid (rRNA) sequence analysis. Unlike *C. violaceum* infection, the patient was successfully treated with a short course of antibiotics, without abscess formation or recurrence. This is the first report to indicate that, although *C. haemolyticum* is phylogenetically related to *C. violaceum*, its impact on humans as an infectious disease may differ completely.

## 2. Case Presentation

The 80-year-old male patient had a known history of hypertension, diabetes mellitus, dyslipidemia, chronic renal disease, and chronic cardiac failure. He attempted to avoid another car while driving and overturned into a rice field 7 days prior to the current admission. He crawled out of his car; however, he collapsed in the rice field, remaining there for several hours. He was transported to the emergency department, and necrotic wounds were identified on his right hand. Subsequently, these wounds were irrigated and sutured. Two days thereafter, the patient presented with fatigue and a loss of appetite. His symptoms gradually worsened to include lethargy and dyspnea. The patient was subsequently admitted to our hospital.

On physical examination, the patient was lethargic, with a blood pressure of 153/80 mm Hg, pulse of 93 beats/min, respiratory rate of 25 breaths/min, oxygen saturation of 96% on room air, and body temperature of 36.6°C. Exudates from the wounds on the dorsal side of the right hand were observed. Breath sounds of the lower pulmonary lobes had decreased bilaterally. Moreover, he had bilateral lower extremity pitting edema. He had a tachycardia, without systolic murmurs. On palpation, his abdomen was soft, without tenderness.

Laboratory investigations were performed. The complete blood count revealed leukocyte and platelet counts of 9.9 × 10^9^/L and 247 × 10^9^/L, respectively, in addition to C-reactive protein and hemoglobin levels of 8.96 mg/dL and 9.4 g/dL, respectively. Renal functions test revealed blood urea nitrogen and creatinine levels of 23 and 2.01 mg/dL, respectively. Liver function tests revealed levels of alanine and aspartate aminotransferases and albumin of 19, 28 U/L, and 3.2 g/dL, respectively. The B-type natriuretic peptide measured 891.2 pg/mL. The serum glucose was 175 mg/dL, and the hemoglobin A1c measured 8.3%.

Chest radiography revealed bilateral pleural effusions with cardiomegaly. A provisional diagnosis of cellulitis of the right hand, complicated by the exacerbation of cardiac failure, was made. Subsequently, two sets of blood cultures were obtained. Empirical antibiotic piperacillin–tazobactam was administered. Diuretic treatment was initiated for the cardiac failure. Three days postadmission, the blood culture revealed a Gram-negative rod. The organism was identified as *C. violaceum* by WalkAway 40 SI (Beckman Coulter, Brea, CA, USA). Most beta-lactam antibiotics, including cefepime, ampicillin–sulbactam, and piperacillin–tazobactam, were inactive against this bacterium. However, this bacterium was sensitive to imipenem, meropenem, ciprofloxacin, levofloxacin, minocycline, doxycycline, and trimethoprim–sulfamethoxazole. Based on these susceptibility results, the piperacillin–tazobactam was switched to meropenem. Abdominal and pelvic ultrasonography revealed no foci of infection, such as abscesses. Sputum culture revealed the presence of normal flora.

**Figure 1:**
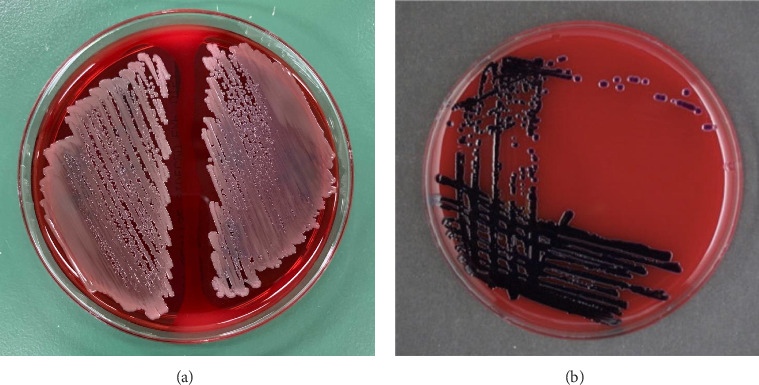
Colonies grown on sheep blood agar plates are shown. (a) Our case of *Chromobacterium haemolyticum* is depicted. The colonies reveal the absence of violet pigmentation with β-hemolysis. (b) *Chromobacterium violaceum* colonies reveal deep violet pigmentation with nonhemolysis.

When the isolated organism was identified as *C. violaceum*, the colonies grown on the sheep blood agar plates were gray and nonpigmented, exhibiting beta-hemolysis (Figure 1). These findings were inconsistent with the features of *C. violaceum.* Matrix-assisted laser desorption ionization time-of-flight mass spectrometry (MALDI Biotyper, MBT Compass library Ver.9.0.0.0, 8468 MSPs, Bruker Daltonics GmbH, Bremen, Germany) was performed; however, the results were inconclusive due to low score values. Homology analysis of the 16S rRNA sequence involving 1458 base pairs of the organism was performed, using the basic local alignment search tool. This demonstrated that the sequence shared the greatest similarities with that of *C*. *haemolyticum* MDA0585^T^ at 99.38% and consecutively with that of *Chromobacterium rhizoryzae* LAM1188^T^ at 99.30%. Finally, we confirmed the organism to be *C. haemolyticum* because *C*. *rhizoryzae* produces tan-colored colonies and is oxidase-negative [12].

The patient's general condition improved after antibiotic and diuretic treatment. His cellulitis improved. The repeated blood culture results were negative. On day 11 postadmission, he was discharged home with oral minocycline, to complete a total of 14 days of effective antibiotic treatment. At the 1-year follow-up, the patient was still in good health with no recurrence of infection.

## 3. Discussion

*C. haemolyticum* was first identified in 2008 by Han et al. [6], who investigated a nonpigmented species of the genus *Chromobacterium* in clinical samples. Prior to its discovery, *C. haemolyticum* was thought to be the same as *C. violaceum*; however, the presence of this novel strain was suggested by the fact that *C. violaceum* produces a violet pigment that contrasts with *C. haemolyticum*. They discovered that *C. haemolyticum* was phylogenetically closely related to *C. violaceum.* However, *C. haemolyticum* has been identified as a different organism due to the lack of colony pigmentation, the ability to hemolyze sheep blood, and differences in several biochemical reactions. The identification of *C. haemolyticum* is important because current automated microbiological identification systems have misidentified *C. haemolyticum* as *C. violaceum*, which occurred in our case.

In previous cases documenting *C. haemolyticum*, this bacterium was not accurately identified, and the authors have highlighted the principal findings differentiating *C. haemolyticum* from *C. violaceum*, which is characterized by colony pigmentation and hemolysis [4, 5, 7–11]. *C. violaceum* is usually present as violet-pigmented colonies because of the production of violacein, without hemolysis occurring in sheep blood agar cultures. In contrast, *C. haemolyticum* is nonpigmented and does not produce violacein, with beta-hemolysis occurring in sheep blood agar cultures [6]. Differences exist in many of the biochemical reactions of the species belonging to the genus *Chromobacterium*; nonetheless, only *C. violaceum* is available for species identification in this genus, using the mass spectrometry database [9]. Therefore, 16S rRNA sequencing is required for definitive diagnosis of *C. haemolyticum* infection. If the isolated organism is identified as *C. violaceum*, however is phenotypically mismatched, clinicians should be aware that the isolated organism may be *C. haemolyticum* instead [8].

Apart from the microbiological features of *C. haemolyticum*, the overall clinical picture of *C. haemolyticum* infection remains unclear owing to its rarity. *C. haemolyticum* and *C. violaceum* are the only causative pathogens of human infections belonging to the genus *Chromobacterium*. However, the clinical similarities and differences between *C. haemolyticum* and *C. violaceum* infections have not received much attention and have been considered similar. *C. violaceum* infection typically occurs after exposure to soil and water [13]. The most prominent feature of this infection is its severity, which rapidly induces acute progression and multiple organ abscesses, resulting in fatal sepsis [13]. Additionally, *C. violaceum* is resistant to commonly used beta-lactam antibiotics and tends to relapse posttreatment, with a reported relapse rate of 6.6% [14]. Relapses are postulated to occur because of residual suppurative foci [15]. Thus, chronic suppression with oral antibiotics is recommended for 2–3 months to prevent relapse [14]. Due to its severity, rapid progression, tendency to develop abscesses, drug resistance, and high recurrence rate, the mortality rate of *C. violaceum* infection is reportedly 35%–53% [13].

Compared to *C. violaceum* infection, *C. haemolyticum* infection is extremely rare; only eight clinically relevant cases have been reported to date including ours [4, 5, 7–11] (Table 1). The diagnostic presentations include necrotizing fasciitis [4], proctocolitis [5], pneumonia [7, 9], meningitis [10], bacteremia [4, 8–11], and cellulitis in one, one, two, one, six, and two patients, respectively [11], some of which overlapped. These cases have been reported in the United States, Japan, and Thailand [4, 5, 7–11]. Six of the eight patients (75%) had known experiences of aquatic exposure and developed infections, including bacteremia, necrotizing fasciitis, and meningitis [4, 7, 9–11]. The findings of severe *C. haemolyticum* infections after exposure to contaminated aquatic environments are very similar to those of *C. violaceum* infections; nonetheless, the outcomes and recurrence rates differ between the two species. Compared with the mortality rate of *C. violaceum*, only one case (13%) of *C. haemolyticum* infection resulted in mortality [10]. One patient with alcohol dependence had neck pain due to a cervical fracture secondary to head trauma, which obscured the diagnosis of meningitis, resulting in delayed initiation of antibiotics. All other patients with *C. haemolyticum* infections survived [4, 5, 7–9, 11], including older adults with multiple comorbidities who developed bacteremia [9]. Remarkably, none of the eight patients (100%) developed abscesses; and four of the eight patients who were successfully cured had not been treated with chronic suppressive antibiotics [5, 7, 11]. Recurrence of infection in the short term has not been reported in these cases. *C. haemolyticum* infection did not recur in our patient during the long-term 1-year follow-up period (Table 2).

Clinicians should be aware that if an isolated organism is originally identified as *C. violaceum*, however is phenotypically mismatched with colony non-pigmentation and beta-hemolysis, the organism may potentially be *C. haemolyticum*. *C. haemolyticum* infections can develop into severe sepsis, postexposure to contaminated water, similar to *C. violaceum*. However, mortality, abscess formation, and recurrence rates are lower in this species than in *C. violaceum*, and chronic broad-spectrum antibiotic suppression may not be required, potentially avoiding unnecessary antibiotic use and preventing multidrug resistance.

## Figures and Tables

**Table 1:** Clinical characteristics of patients with *Chromobacterium haemolyticum* infection reported to date.

No.	Age	Sex	Diagnosis	Positive culture	Incident	Presumed source of organism	Comorbidity	Abscess formation	Antibiotics	Effective antibiotics	Outcome	Recurrence	Country	Reference
1	26	M	Necrotizing fasciitis, bacteremia	Blood	Road accident	River	None	No	Ampicillin–sulbactam + Minocycline -> Ceftazidime -> Ciprofloxacin + Gentamicin -> Ciprofloxacin	6 weeks	Survived	None for 6 months	Japan	[4]

2	4	F	Proctocolitis	Stool	—	—	—	No	Ceftriaxone -> Ciprofloxacin + Metronidazole	10 days	Survived	None for 6 weeks	Thailand	[5]

3	69	M	Pneumonia	Sputum	Water aspiration	Runoff water	Hypertension, chronic kidney disease, cerebral infarction	No	Ampicillin–sulbactam -> Meropenem -> Piperacillin–tazobactam	11 days	Survived	Transferred to another hospital	Japan	[7]

4	11	M	Catheter-associated bacteremia	Blood	—	—	Congenital heart disease /hardware installation, Kingella endocarditis	No	Meropenem + Gentamicin + Trimethoprim–sulfamethoxazole -> Levofloxacin	8 weeks	Survived	None for 3 months	United States of America	[8]

5	70s	M	Pneumonia, bacteremia	Sputum, blood	Near-drowning	River	Hypertension, diabetes mellitus, benign prostatic hyperplasia	No	Meropenem + Levofloxacin -> Ceftazidime + Levofloxacin -> Levofloxacin	11 weeks	Survived	—	Japan	[9]

6	73	M	Meningitis, bacteremia	Spinal fluid, blood	Head trauma	Canal	Hypertension, hyperuricemia, alcoholic hepatitis	No	Meropenem + Vancomycin	4 days	Demised	—	Japan	[10]

7	50s	M	Cellulitis, bacteremia	Blood	Skin injury	Hot spring	—	No	Cefalexin -> Cefepime + Metronidazole -> Levofloxacin -> Meropenem -> Trimethoprim–sulfamethoxazole	17 days	Survived	—	United States of America	[11]

Our case	80	M	Cellulitis, bacteremia	Blood	Skin injury	Rice field	Hypertension, dyslipidemia, diabetes mellitus, heart failure, chronic kidney disease	No	Piperacillin–tazobactam -> Meropenem -> Minocycline	2 weeks	Survived	None for 1 year	Japan	—

**Table 2:** Differences between *Chromobacterium haemolyticum* and *Chromobacterium violaceum* in microbiological and clinical features.

	*Chromobacterium haemolyticum*	*Chromobacterium violaceum*
*Microbiological features*		
Colony pigmentation	Nonpigmentation	Violet pigmentation
Hemolysis	Beta-hemolysis	Nonhemolysis

*Clinical features*		
Incidence	Extremely rare: 7 cases	Rare
Environment	Tropical/subtropical ecosystems	Tropical/subtropical ecosystems
Source of infection	Contaminated soil and water	Contaminated soil and water
Progression	Acute progression with sepsis	Acute progression with sepsis
Abscess formation	No	Multiple organ abscesses
Recurrence	No	Yes: 6.6%
Susceptibility	Resistant to common beta-lactams	Resistant to common beta-lactams
Duration of antibiotics	May be cured with short duration	Chronic suppression for 2-3 months is recommended
Mortality	May not be poor ≤ 13%	Poor: 35%–53%

## Data Availability

The data used to support the findings of this study are included within the manuscript.
